# Culturing of respiratory viruses in well-differentiated pseudostratified human airway epithelium as a tool to detect unknown viruses

**DOI:** 10.1111/irv.12297

**Published:** 2014-12-04

**Authors:** Seyed Mohammad Jazaeri Farsani, Martin Deijs, Ronald Dijkman, Richard Molenkamp, Rienk E Jeeninga, Margareta Ieven, Herman Goossens, Lia van der Hoek

**Affiliations:** a Laboratory of Experimental Virology, Department of Medical Microbiology, Center for Infection and Immunity Amsterdam (CINIMA), Academic Medical Center, University of AmsterdamAmsterdam, The Netherlands; b Tehran University of Medical SciencesTehran, Iran; c Laboratory of Clinical Virology, Department of Medical Microbiology, Center for Infection and Immunity Amsterdam (CINIMA), Academic Medical Center, University of AmsterdamAmsterdam, The Netherlands; d Department of Medical Microbiology, Vaccine and Infectious Disease Institute, Universiteit Antwerpen, University Hospital AntwerpAntwerp, Belgium

**Keywords:** Airway epithelial cultures, influenzavirus B, respiratory viruses, VIDISCA-454, virus discovery

## Abstract

**Background:**

Currently, virus discovery is mainly based on molecular techniques. Here, we propose a method that relies on virus culturing combined with state-of-the-art sequencing techniques. The most natural *ex vivo* culture system was used to enable replication of respiratory viruses.

**Method:**

Three respiratory clinical samples were tested on well-differentiated pseudostratified tracheobronchial human airway epithelial (HAE) cultures grown at an air–liquid interface, which resemble the airway epithelium. Cells were stained with convalescent serum of the patients to identify infected cells and apical washes were analyzed by VIDISCA-454, a next-generation sequencing virus discovery technique.

**Results:**

Infected cells were observed for all three samples. Sequencing subsequently indicated that the cells were infected by either human coronavirus OC43, influenzavirus B, or influenzavirus A. The sequence reads covered a large part of the genome (52%, 82%, and 57%, respectively).

**Conclusion:**

We present here a new method for virus discovery that requires a virus culture on primary cells and an antibody detection. The virus in the harvest can be used to characterize the viral genome sequence and cell tropism, but also provides progeny virus to initiate experiments to fulfill the Koch's postulates.

## Introduction

The discovery of new viruses has been boosted in the last decade by high-throughput sequencing methods. These techniques can generate tens of thousands of sequence reads directly from a clinical sample, and sequence alignment tools subsequently can reveal the presence of previously unknown viruses. The main limitation of these viral metagenomics techniques is that the detection of sequence reads derived from a viral genome does not necessarily indicate that the virus is pathogenic, in the absence of information on phenotypic properties such as infectivity, cell tropism, and the ability to induce the immune system.^1^

Once a new virus is identified, the fulfillment of Koch's postulates is needed to establish the role of the virus in disease. A virus culture stage is thus needed to obtain relatively pure virus stocks for inoculation in an animal model. Virus culturing remains a major bottleneck. In the 20th century, virus research and identification were for a large part limited to those agents that could be cultured in conventional cell lines. More recently, powerful sequencing methods allow the identification of new viruses in clinical samples, for which a virus culture as amplification step is no longer required. The downside is that without a virus culture, it is not possible to formally fulfill the Koch's postulates. As a result, one can describe, at most, a disease association, either by a higher viral prevalence in infected subjects compared to controls or by seroconversion to the agent during the course or following the disease.^2^

Well-differentiated pseudostratified airway epithelium is formed by culturing of primary human airway epithelial cells (HAE) at an air–liquid interface. The morphology and functionality of the cells resemble those of the human airways, and this system has been used to culture a wide range of respiratory viruses, for example, influenzavirus A,^3^ parainfluenza virus,^4^ respiratory syncytial virus,^5^ adenovirus,^6^ and severe acute respiratory syndrome coronavirus.^7^ Furthermore, some of the viruses which have recently been described can be cultured on these cells, whereas all regular cell lines are not permissive.^8^–^10^ These results collectively suggest that the HAE cultures are a very promising tool for universal respiratory virus discovery.

The combination of these powerful techniques, virus HAE cultures for virus isolation and next-generation sequencing to detect the viral genome, might be ideal for future virus discovery programs. There is, however, one pitfall with HAE cultures. Even with a fast replicating respiratory virus, a cytopathic effect is rarely observed. Some influenzavirus A strains cause cell death, but the majority of infections do not change the epithelial layer. Thus, HAE should be combined with a virus detection, for which we propose immunostaining with convalescent serum collected from the same patient obtained a few weeks after the respiratory infection. This serum will likely contain substantial antibody titers against the virus that caused the respiratory illness a few weeks earlier.

In this proof of principle study, we tested the combination of (1) replication of an unknown respiratory virus on HAE cell cultures, followed by (2) immunostaining with the patient's serum, and (3) unbiased detection of the infecting virus by a metagenomics virus discovery tool: VIDISCA-454 (virus discovery cDNA-AFLP combined with Roche 454 high-throughput sequencing). The latter is an amplification technique developed in our laboratory that allows sequencing of both RNA and DNA viruses independent of the genome sequence.^11^–^15^ Three respiratory samples—anonymized for the respective infecting agent as determined by routine diagnostics—were included in this study. In all three cases, the virus could be cultured, detected with the patient's own antibodies, and identified with VIDISCA-454.

## Materials and methods

### Clinical materials

Three respiratory samples (Copan nasal swabs in universal transport medium) collected from patients with lower respiratory tract illness via the GRACE European Network of Excellence^16^ were tested blindly. Sample S2705 was collected from a patient in Bialystock (Poland), I2125 from Lodz (Poland), and E1517 from Mataro (Spain). Serum was collected 5 weeks after the acute phase of lower respiratory tract illness from the same patients.

### Ethical approval

Ethics review committees in each country approved the study: Mataro (Spain): Comitè d'Ètica d'Investigació Clínica (CEIC) del Consorci Sanitari del Maresme; Lodz and Bialystok (Poland): Komisja Bioetyki Uniwersytetu Medycznego W Lodzi; written informed consent was provided by all study participants.

### Human airway epithelial cell culture

Normal primary human bronchial epithelial cells (HBEpC) were isolated from patients (>18 years old) who underwent bronchoscopy and/or surgical lung resection in their diagnostic pathway for any pulmonary disease. This was carried out in accordance with local regulations from the Academic Medical Center, the Netherlands. Pathologically examined bronchial segments were incubated for 48 hours at +4°C in minimal essential medium (MEM; Sigma, St. Louis, MO, USA) supplemented with a mixture of protease XIV-DNase I (Sigma) and the following additives (Sigma): penicillin G sulfate (100 units/ml), streptomycin sulfate (100 μg/ml), amphotericin B (1·25 μg/ml), gentamicin (50 μg/ml), and nystatin (100 units/ml). After cell dissociation, the HBEpC were maintained as a monolayer in bronchial epithelial cell serum-free growth medium (BEGM), which is LHC basal medium (Invitrogen, Carlsbad, CA, USA) supplemented with the required additives described by Fulcher *et al*.^17^ BEGM was refreshed at 2- or 3-day intervals. When reaching 75% confluence, cells were dissociated with 2 ml of TrypLE Express enzyme (Invitrogen) and diluted in air–liquid interface medium,^17^ which is a mixture of LHC basal medium (Invitrogen) and Dulbecco's modified Eagle's medium (Invitrogen) supplemented with the required additives^17^ (Sigma). A total of 8 × 10^4^ cells were seeded on type IV collagen (Sigma)-coated 12-well ThinCerts with a 0·4-μm pore size (Greiner Bio-One, Frickenhausen, Germany). Medium was renewed every 2 or 3 days. When cultures reached full confluence, the cells were exposed to air. Cultures on the air–liquid interface were maintained in 12-well deep-well plates (Greiner Bio-One) for 21 days to let the cells differentiate into pseudostratified human airway epithelial cell cultures. Medium from the basolateral compartment was renewed every 7 days, and the apical surface was washed every 2 days with Hanks' balanced salt solution (HBSS) (Invitrogen). Prior to the experiments, all cultures were maintained at 37°C in a 5% CO_2_ incubator.

### Inoculation of HAE

Prior to infection, the apical surfaces of HAE cells were rinsed three times with HBSS and then inoculated on the apical surface with 200 μl of 1:2-diluted respiratory sample. Following 2 hours incubation at 34°C in a 5% CO_2_ incubator, the unbound virus was removed by rinsing the apical surface with 500 μl HBSS for 10 min at 34°C, and the HAE were maintained at an air–liquid interface for the remainder of the experiment at 34°C. Harvests were collected after 24, 48, 72, 96, and 120 hours post-inoculation from both the apical and basolateral sides, except for sample E1517 for which the last harvests were collected at 96 hours post-inoculation. Apical washing and harvesting were performed by adding 200 μl HBSS to the apical surface and incubation for 10 minutes at 34°C in a 5% CO_2_ incubator, followed by the removal and storage of the 200 μl HBSS from the apical surface.

### Confocal microscopy analysis

Ninety-six or 120 hours post-infection cultures were used for immunostaining. These time-points were chosen to be sure to be able to detect infected cells even of viruses that replicate slower that the average respiratory viruses (average peak at 72 to 96 hours post-infection^8^,^9^). The apical and basolateral sides were rinsed with phosphate-buffered saline (PBS), followed by fixation with freshly prepared 4% paraformaldehyde (PFA; FormaFix) for 30 minutes at room temperature. The apical and basolateral sides were subsequently washed three times with 500 μl PBS, and confocal staining buffer (PBS supplemented with 50 mm NH_4_Cl, 0·1% saponin and 2% BSA (IgG-free)) was added to both sides. After subsequent washing with confocal staining buffer, the cells were incubated at the apical side with the autologous convalescent serum of the patient (250 μl, 1:25 dilution in confocal staining buffer) and mouse monoclonal anti-β-tubulin IV (1:400) (Sigma) for 120 minutes at room temperature. Cells are subsequently washed with confocal staining buffer followed by incubation with Donkey-derived Dylight 488-labeled anti-mouse IgG(H+L) and Dylight 594-labeled anti-human IgG(H+L) (both diluted 1:200 in confocal staining buffer, Jackson Immunoresearch) for 1 hour at RT. Cells are washed with confocal staining buffer and finally with PBS to remove the saponin. The filter with cells was subsequently excised from the insert and placed on a glass slide with mounting medium (Molecular Probes), apical side upwards, mounting medium, and coverslip added on top. Fluorescence images were acquired using a Leica TCS SP2 AOBS spectral confocal microscope (Wetzlar, Germany). Image capture, analysis, and processing were performed using the Leica Application Suite, Advanced Fluorescence Lite software packages (Leica).

### VIDISCA and Roche Titanium-454 sequencing

VIDISCA-454 was performed as previously described.^12^ In short, virus harvests were centrifuged for 10 min at 10 000 g, and the supernatant was treated with TURBO DNase (2 U/μl Ambion, Austin, TX, USA). Subsequently, nucleic acids were extracted by the Boom method.^18^ rRNA-blocking oligonucleotides were added to prevent amplification of ribosomal RNA, and a reverse transcription reaction with Superscript II (Invitrogen) was performed using non-ribosomal random hexamers.^19^ Subsequently, second-strand DNA synthesis was performed with 5 U of Klenow fragment (New England Biolabs, Ipswich, MA, USA). Double-stranded DNA was purified by phenol/chloroform extraction and ethanol precipitation and digested with Mse I restriction enzyme (New England Biolabs). Adaptors with different Multiplex Identifier sequences (MIDs) were ligated to the digested fragments of the different samples. Before PCR amplification, the fragments were purified with AMPure XP beads (Agencourt AMPure XP PCR, Beckman Coulter, Massachusetts, USA). Next, a 28-cycle PCR with adaptor-binding primers was performed: the program of PCR was as follows: 5 minutes 95°C, and cycles of 1 minute 95°C, 1 minute 55°C, and 2 minutes 72°C, followed by 10 minutes 72°C and 10 minutes 4°C. After purification with AMPure XP beads, the purified DNA was quantified with the Quant-it dsDNA HS Qubit kit (Invitrogen) and diluted to 10^7^ copies/μl. Samples were pooled and Kapa PCR (Kapa Biosystems, Wilmington, MA, USA) was performed to determine the quantity of amplifiable DNA in each pool. Subsequently, the Bioanalyser (hsDNA chip, Agencourt) was used to determine the average nucleotide length of the libraries, and the pools were diluted until 10^6^ copies/μl to be used for a titration (DNA:beads ratio of 0·5:1, 1:1, 2:1 and 4:1) in an emulsion PCR according to the suppliers' protocol (LIB-A SV emPCR kit). Sequencing was performed on a two region GS FLX Titanium PicoTiterPlate (70 × 75) with GS FLX Titanium XLR 70 Sequencing kit (Roche, Mannheim, Germany).

### Sequence analysis

Primer, MID, and ribosomal RNA sequences were trimmed or removed from the sequence reads. Sequences were assembled with CodonCode Aligner software (CodonCode Corporation, Centerville, MA, USA) version 3.5.6. The contig sequences and the unassembled reads were compared with available sequences in Genbank via the BlastN tool using default settings. The blast output was used to create a taxonomic classification of the reads with Megan software version 4.70.4 (University of Tübingen, Tübingen, Germany). The following settings were used: Min Support: 1, Min Score: 50, Min Score/Length: 0·5, Top Percent 100.

## Results

### Virus replication on pseudostratified airway epithelium

**Figure 1:**
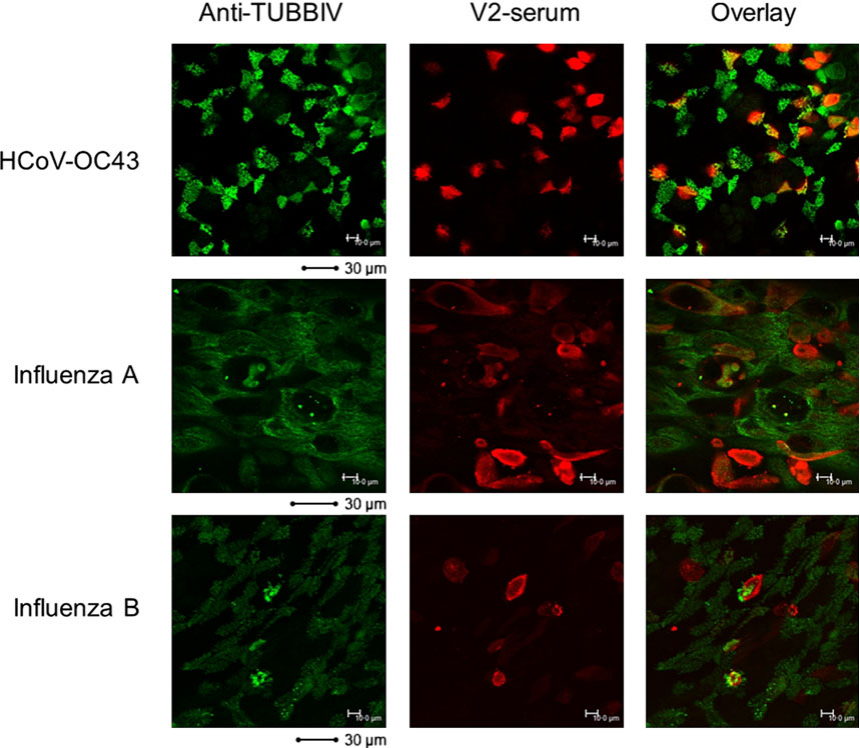
Immunostaining of infected human airway epithelial (HAE) cell cultures with patient sera. Three respiratory samples, S2705 (HCoV-OC43), I2125 (influenzavirus A, H3N2), and E1517 (influenzavirus B), were used to inoculate human airway epithelial (HAE) cell cultures. The cells were fixed 96 or 120 hours post-inoculation with 4% PFA and immunostained with the autologous antibody derived from respective patients (red) and mouse monoclonal anti-ß-tubulin IV (green), and examined by confocal microscopy. The difference in intensity of tubulin staining represents experimental variation in differentiation (S2705 versus E1517) or a difference in focus depth (I2125, below the apical surface). An overlay image was generated to determine the cell tropism of each virus. Bars: 30 μm.

Three respiratory samples were used to inoculate well-differentiated human pseudostratified epithelial cultures. The clinical material was used to inoculate the apical surface of the cultures. After 2 hours, unbound viral particles were removed by washing. The cultures were maintained for 96 to 120 hours, followed by fixation of the cells in 4% PFA. Immunostaining of infected cells was performed with convalescent sera from the corresponding patient obtained approximately 5 weeks after the onset of symptoms, when full recovery of the patient was apparent. In theory, these sera will contain a substantial amount of pathogen-specific immunoglobulins that can recognize epitopes exposed on the virus surface.^20^,^21^ In addition, cells were stained with a monoclonal antibody against a ciliated cell-specific marker (ß-tubulin IV) to identify ciliated cells. For all three cultures, infected cells could be visualized by confocal microscopy (Figure 1). In sample S2705 staining of infected ciliated cells was visible, whereas sample I2125 and E1517 showed staining of ciliated and non-ciliated cells. Furthermore, a strong cytopathic effect was noted for I2125, with detachment of cells from the filter, and cell debris in the apical washing.

### Virus genome characterization

The apical wash obtained at 96 or 120 hours post-inoculation was used as input for VIDISCA-454 virus discovery. From the three culture harvests, a total of 9124 sequence reads were obtained: 3227 from S2705, 2686 from I2125, and 3211 from E1517. An identity search using the BLAST tool from NCBI showed that each sample contained a different respiratory virus: human coronavirus OC43 (sample S2705), influenzavirus A H3N2 (sample I2125) or influenzavirus B (sample E1517). The number of reads was substantial for each virus (in all cases more than 10% of the total reads). More importantly, a large coverage of the genome was obtained (>50% for all viruses, see supplementary data), which is remarkable as only a few thousand sequences were obtained per sample (Table 1).

**Table 1:** Sequence reads in respiratory samples upon human airway epithelial (HAE) culturing

Sample name	Total number of sequence reads	Reads of viral origin (%)	Virus identified	Genome coverage (%)
S2705	3227	10·8%	HCoV-OC43	51·6%
I2125	2686	13·2%	Inf-A (H3N2)	57·5%
E1517	3211	88·5%	Inf-B	81·8%

### Influenzavirus B replication

**Figure 2:**
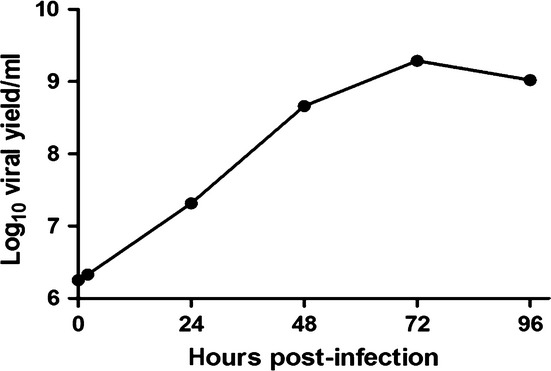
Replication of influenzavirus B in human airway epithelial (HAE) cell culture. Apical harvests of the E1517 culture were collected at 2, 24, 48, 72, and 96 hours post-inoculation. Viral RNA was quantified by real-time PCR as previously described.^33^

The replication of influenzavirus B on HAE is noteworthy. To our knowledge, this is the first description of a spreading infection caused by this virus on HAE. We therefore determined the replication characteristics by quantification of viral RNA in the apical harvests (Figure 2). Virus production can be detected as early as 24 hours post-infection, with a maximum at 72 hours post-infection.

## Discussion

The currently available high-throughput sequencing platforms can generate an impressive amount of sequence information in a short time frame. But the acquisition of essential additional knowledge that is needed to address the pathogenicity of a novel virus is lagging behind.^22^,^23^ The discovery of a novel human virus should preferably be accompanied by serology (seroconversion to the virus between the acute and convalescent phase of the disease), an association with disease (more frequent detection in disease cases compared to healthy controls), and ideally a virus culture system as the first requirement in fulfilling the Koch's postulates. In this study, we present a combination of a virus culture method and a virus discovery tool that allows the identification of respiratory viruses that 1) have elicited an immune response and 2) are able to infect cells that match the affected organ from which the clinical specimens were originally obtained.

Three clinical samples were used to evaluate the combined culture-virus-discovery tool. For all three viruses, staining of the infected HAE cells by convalescent serum from the patients was positive, and the apical washes were investigated by VIDISCA-454, a sequence-independent virus discovery tool that can detect RNA and DNA viruses.^12^ The sequence reads provide a high genome coverage. This provides the opportunity of identifying viruses that are highly divergent from known viruses because the inclusion of a conserved domain is more likely. Of each apical wash, only a few thousand sequence reads were sufficient. The low number of reads needed to identify the cultured virus has the advantage that many samples can be analyzed in parallel in a single 454 run (approximately 150 samples per run), which makes the assay only moderately expensive. One should note, however, that this combined assay is currently not suitable to be used in a high-throughput setting, because of the significant hands-on time and expert experience needed to prepare the HAE cell cultures. On the other hand, the availability of a culture method is enormously beneficial for follow-up studies as it provides the progeny virus material, for example, for inoculation of animals to fulfill the postulates of Koch.

We document the replication of three respiratory viruses in respiratory tract cells, but equally relevant is the use of an equivalent combined system for the discovery of pathogenic gastrointestinal viruses. Fecal samples contain a whole range of non-relevant viruses—many still unknown—that either originate from food or from the collection of phages that infect intestinal bacteria. Advanced intestinal epithelial culture systems have been developed of which the Human Intestinal Organoid system is promising for virus replication.^24^ This could be combined with staining and virus discovery to identify only those viruses that infect human cells in the intestine. We recently published a virus discovery tool that relies on antibody capture,^25^ thereby circumventing the problem that food-related viruses can be mistaken for human pathogens. Adding a virus culture step would add even more weight to such a discovery because bacteriophages and food-infecting viruses are incapable of infecting intestinal epithelial cells.

An additional advantage of immunostaining to identify virus-positive cultures is that the cell tropism of the virus can be determined at the same time. We observed infection of ciliated cells by HCoV-OC43, and infection of ciliated and non-ciliated cells in influenzavirus A infection, in accordance with the literature.^26^,^27^ Influenzavirus B replicated in ciliated and non-ciliated cells, which has not been described previously. The human influenzavirus A H3N2 and B HA proteins are structurally very similar and both preferentially bind to α^2^,^6^-linked sialic acids,^28^,^29^ which is in accordance with the cell tropism of both viruses.

The choice for well-differentiated pseudostratified human airway epithelial cells was based on our in-house experience.^8^,^9^,^26^ The majority of respiratory viruses can replicate in these cultures, even the ones that cannot be cultured on traditional cell lines (e.g., human bocavirus and human coronavirus HKU1^8^,^9^). The procedure to culture these differentiated cells has been described in detail, but handling of the cells requires technical experience and relatively much hands-on time and is therefore not routinely available in most laboratories. An alternative may be to obtain HAE from commercial suppliers (e.g., 3D human upper airway epithelia from MucilAir™, Epithelix, Geneva, Switzerland),^30^,^31^ but we have not compared these cultures to ours.

We did not perform a negative staining control (infection of cells, but leaving out the convalescent serum) to check whether the procedure would give false-positives. We have performed this control, however, in other studies, and staining of infected and uninfected cells is distinctively negative when no primary antibody is added.^26^ Another control, no infection of cells but incubation with the convalescent serum, was also not included here. This control is, however, not needed in virus discovery. The ultimate identification of a virus is via the VIDISCA-454 sequence reads. A false-positive staining cannot result in a false-positive virus identification or discovery.

The method may not work if the staining with convalescent serum yields no signal. This can occur when 1) there is only IgM or IgA and no IgG yet, and the secondary antibody only recognizes IgG; or 2) the only response to an infection occurs in the mucosa via IgA. The latter issue could theoretically be addressed if a later respiratory sample is available, and this material subsequently used as source for primary antibodies.

One limitation of the combined discovery tool is that most patient studies do not collect convalescent serum after an infection. In case this material is lacking, there is the possibility to use concentrated immunoglobulins from multiple donors (IVIg; Nanogam, Sanquin B.V.). This pool will be able to detect viruses that are common in the population yet have not been discovered. This was the case for rhinovirus C, HCoV-HKU1, HCoV-NL63, human bocavirus, etc. The pooled immunoglobulins could also recognize emerging viruses, like SARS-CoV and/or MERS-CoV, as it has been shown that antibodies directed to the common cold coronaviruses may display some cross-reactivity with newly emerging coronaviruses.^32^

In conclusion, we present a combined virus discovery approach to exclusively identify viruses that infect human cells and that have elicited an immune response. Both are strong indicators that the identified agent is the causative agent of the disease.

## Supporting Information

**Figure S1.** Supplimentary 1. Influenza A, influenza B and HCoV-OC43 Alignments.
