# Supplementary material for: Culturing of respiratory viruses in well-differentiated pseudostratified human airway epithelium as a tool to detect unknown viruses
Source: Influenza Other Respir Viruses. 2014 Dec 4;9(1):51–7. doi: 10.1111/irv.12297 (PMC4280819; doi:10.1111/irv.12297)
Supplement: Supplementary file 1 — Figure S1. Supplimentary 1. Influenza A, influenza B and HCoV-OC43 Alignments. [file irv0009-0051-sd1.doc]

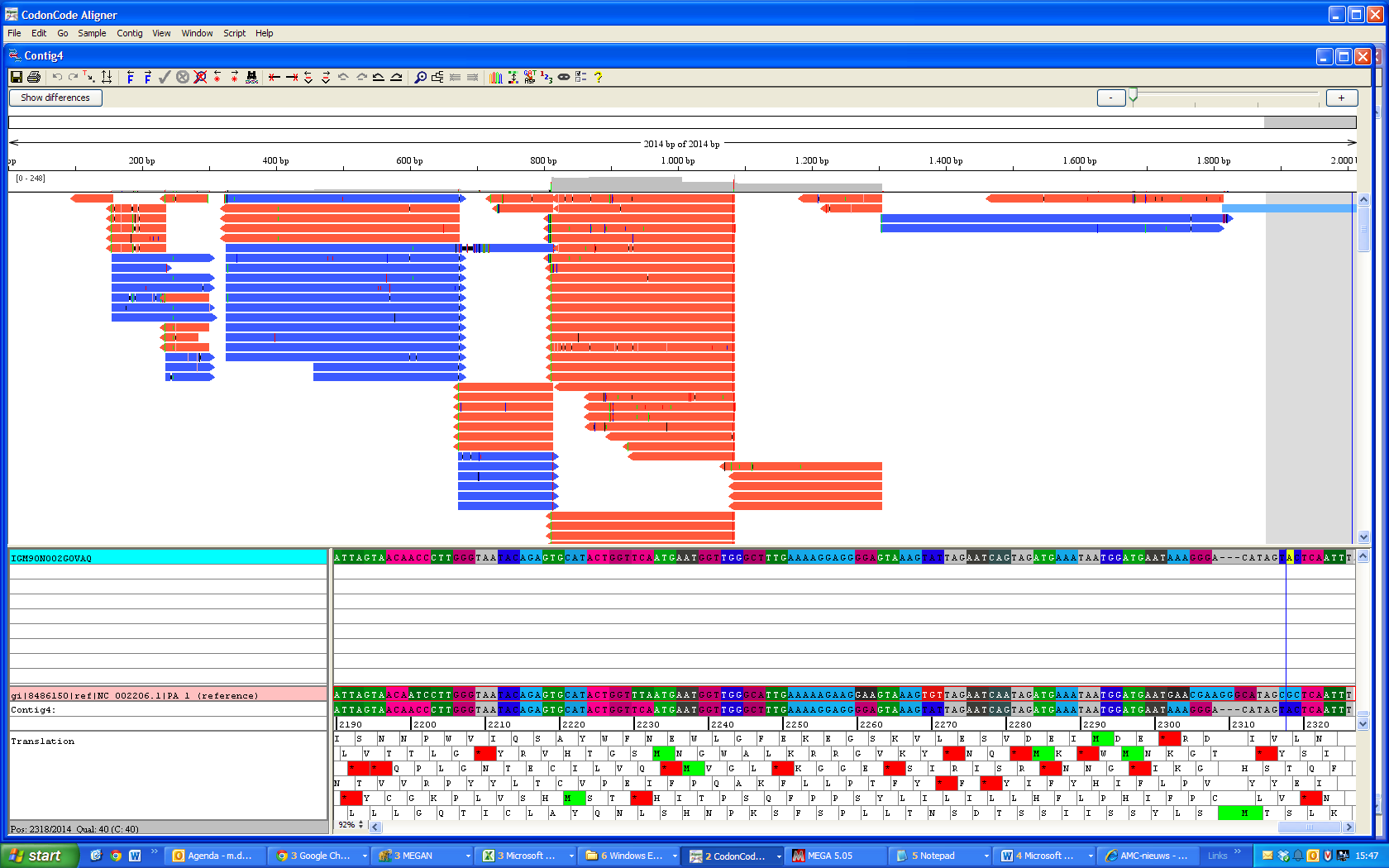


E1517 Influenza B Polymerase PA


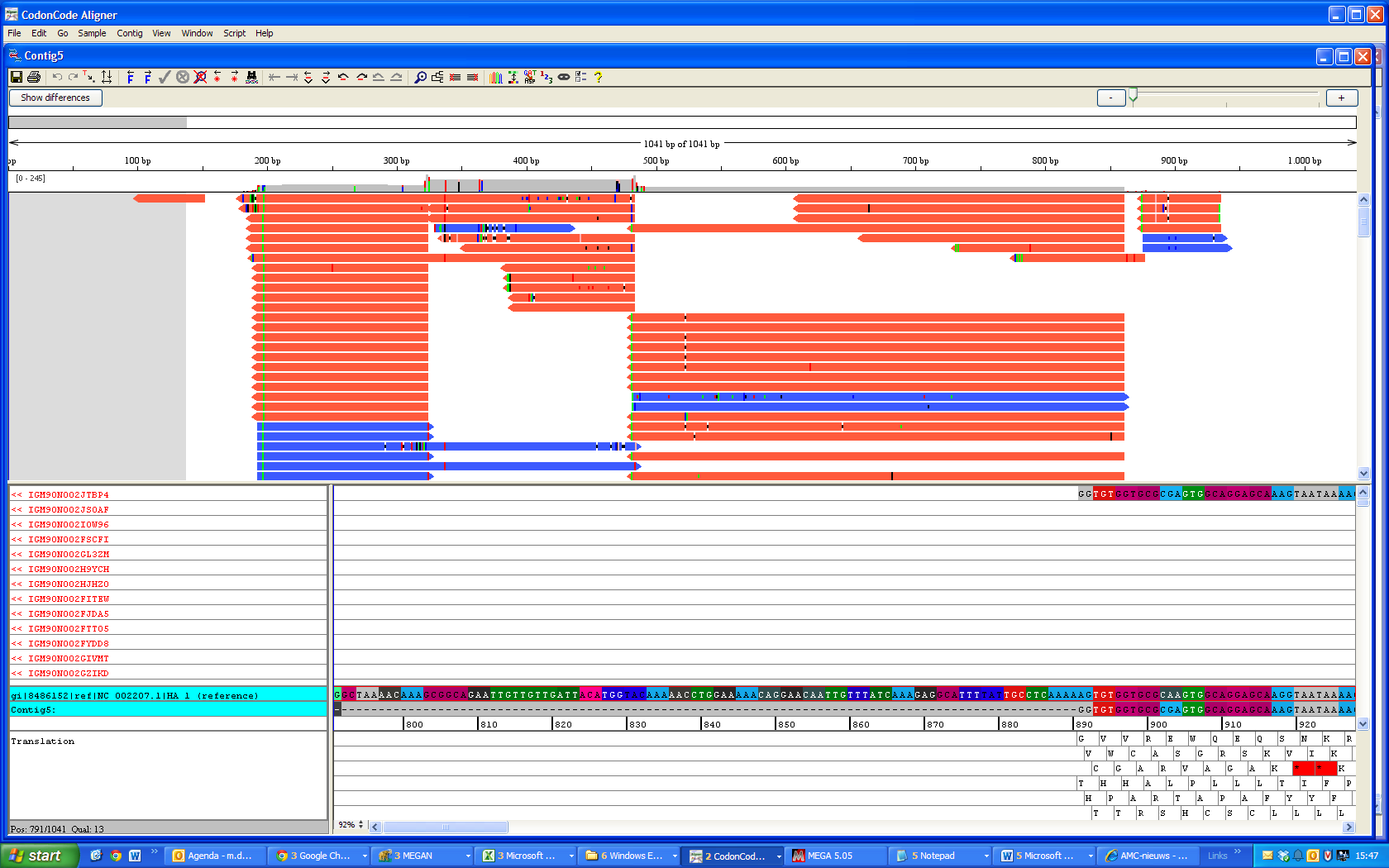


E1517 Influenza B Hemagglutinin


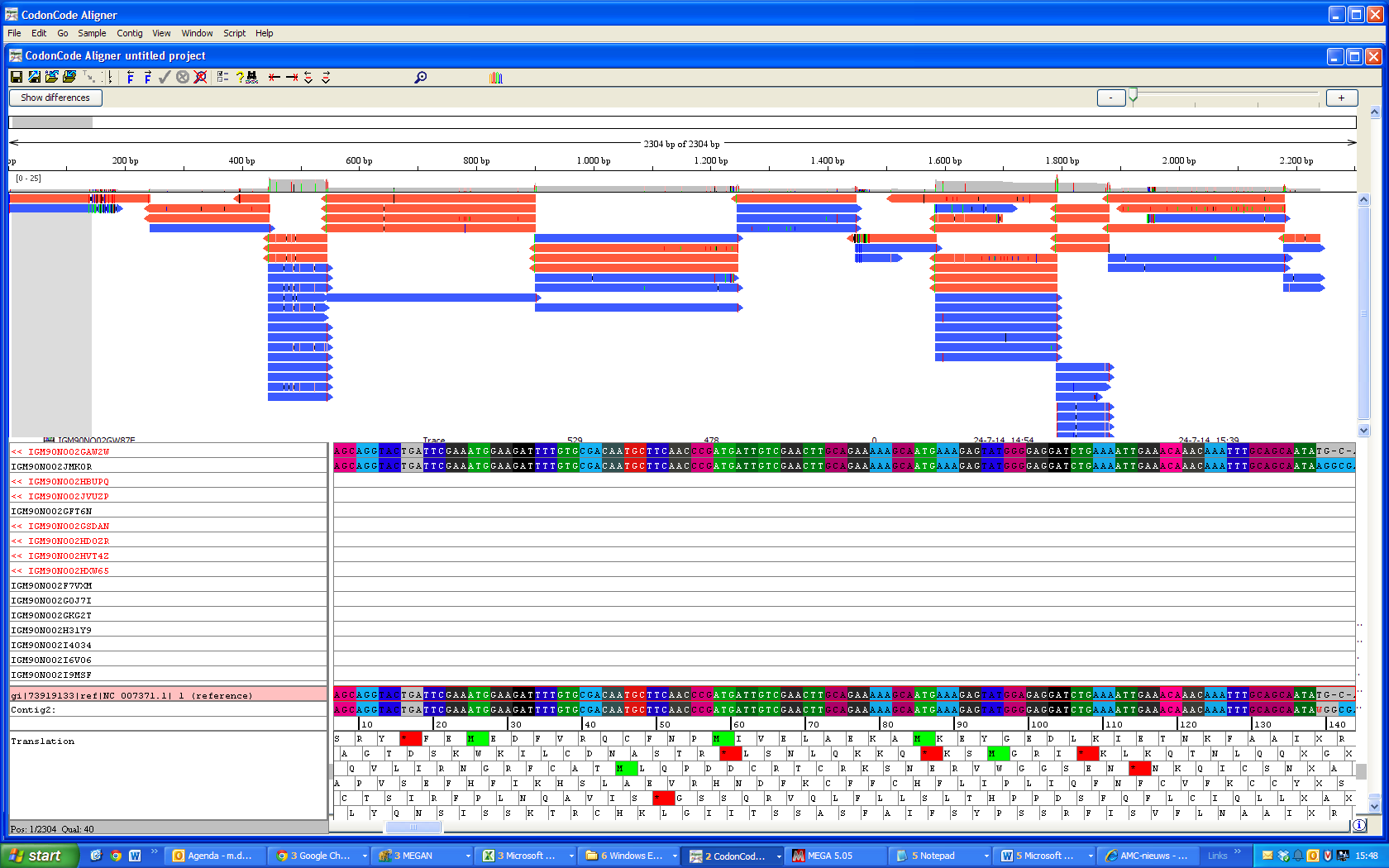


I2125 Influenza A (H3N2) Polymerase PA


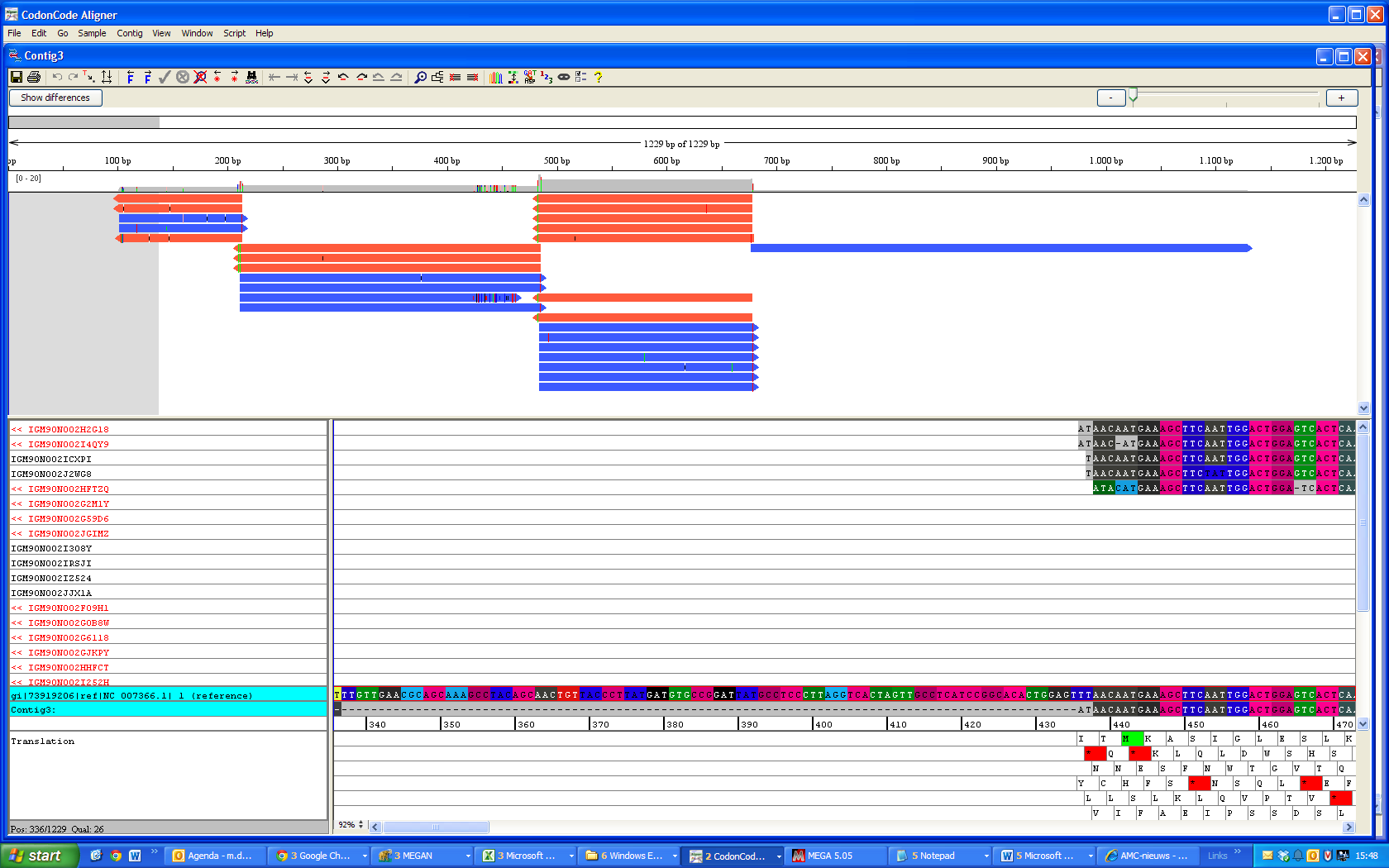


I2125 Influenza A (H3N2) Hemagglutinin


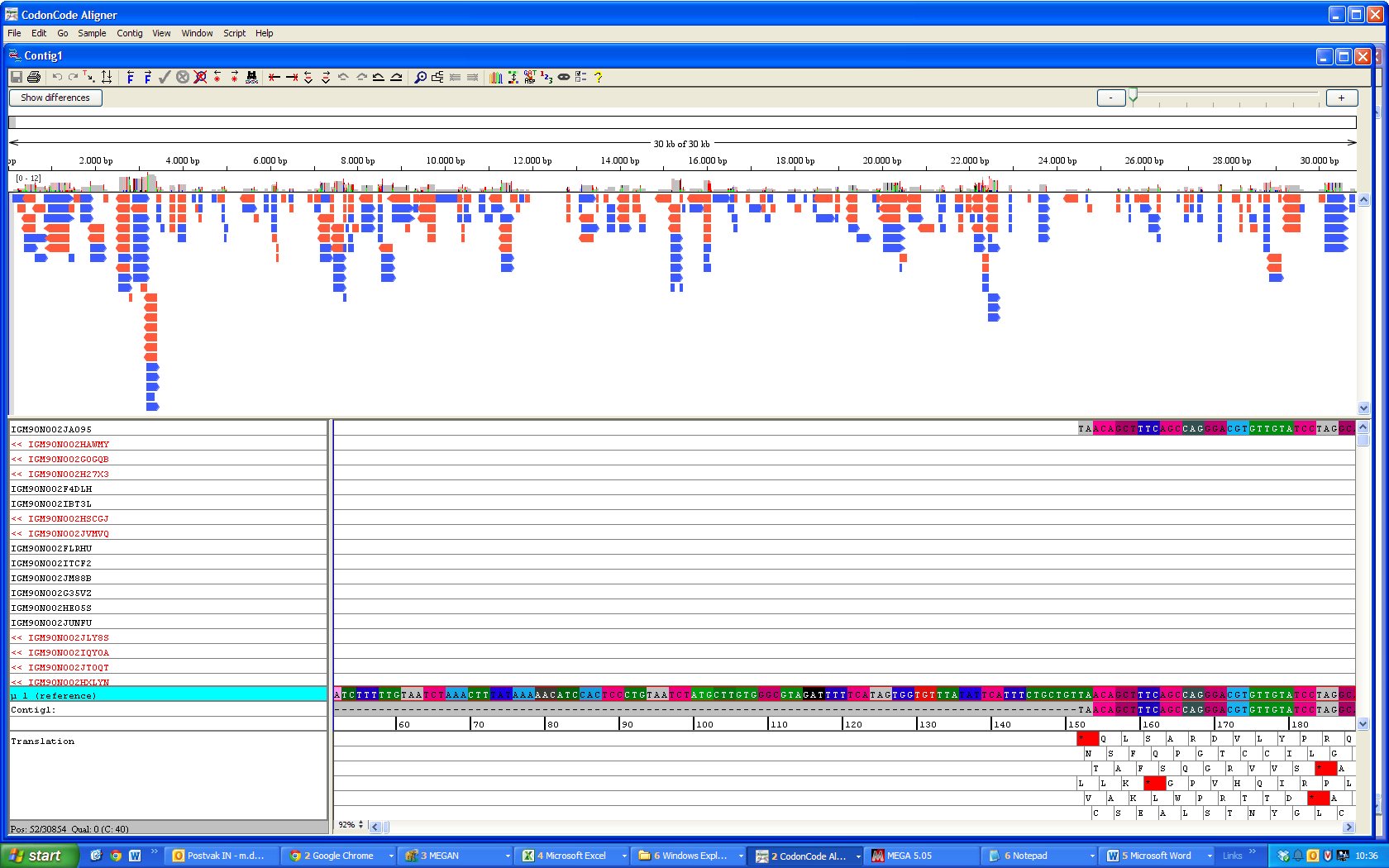


S2705 Human Coronavirus OC43
